# Electroacupuncture combined with subanesthetic alfaxalone as a novel target for alleviating neuropathic pain

**DOI:** 10.1097/PR9.0000000000001296

**Published:** 2025-09-03

**Authors:** Lingling Liu, Mahmoud M. Abouelfetouh, Yi Ding, Lei Qianghui, Rui Sun, Eman Salah, Sha Nan, Mingxing Ding, Yuzhen Song

**Affiliations:** a Henan University of Animal Husbandry and Economy, Zhengzhou, Henan Province, China; b College of Veterinary Medicine, Huazhong Agricultural University, Wuhan, Hubei Province, China; c Department of Surgery, Radiology and Anesthesiology, Faculty of Veterinary Medicine, Benha University, Moshtohor, Egypt; d College of Basic Medical Sciences, Naval Medical University, Shanghai, China; e Department of Pharmacology, Faculty of Veterinary Medicine, Benha University, Moshtohor, Egypt

**Keywords:** Electroacupuncture, Alfaxalone, Neuropathic pain, Gamma-aminobutyric acid (GABA), Diazepam-binding inhibitor (DBI)

## Abstract

**Introduction:**

Neuropathic pain severely affects patients' quality of life. Limited treatments offer relief but often involve long-term use and side effects.

**Objectives:**

This study aimed to explore the electroacupuncture (EA) and subanesthetic alfaxalone (ALF) combination as a novel therapeutic substitute for the treatment of neuropathic pain in a spared nerve injury (SNI) mouse model.

**Methods:**

Sixty healthy C57BL/6 male mice were randomized into 5 equal-sized groups: sham (n = 12) group, spared nerve injury (SNI; n = 12) group, SNI-ALF (n = 12), SNI-EA (n = 12), and SNI-EA-ALF (n = 12). The SNI mice received the treatment regimens at 8 days postoperatively once every 2 days for 7 treatments in total. The mechanical and thermal pain thresholds were tested after each treatment. Spinal cord samples were collected after the fourth and seventh treatments for detection of diazepam-binding inhibitor (DBI) and γ-aminobutyrate A1 isoform receptor (GABAA1).

**Results:**

Mice in the SNI-EA-ALF group showed a significant increase in mechanical and thermal pain thresholds as compared to those in the SNI-ALF and SNI-EA groups during the treatment period (*P* < 0.05). A significant increase in GABAA1 expression was observed after the fourth and seventh treatments in the SNI-EA-ALF group compared to the SNI-ALF and SNI-EA groups (*P* < 0.05). In addition, the DBI expression level was significantly lower in the SNI-ALF-EA group than the SNI, SNI-ALF, and SNI-EA groups (*P* < 0.05).

**Conclusion:**

Our results support the use of EA, combined with ALF, to synergistically relieve pain in preclinical models of NP.

## Abstract 2

Electroacupuncture and alfaxalone improve the expression of GABA A receptor via downregulating diazepam-binding inhibitor in the spinal cord and potentiates the antineuropathic pain effect.

## 1. Introduction

Neuropathic pain (NP) is a significant medical and socioeconomic burden worldwide.^3^ It affects up to 14.6% of the general population in the United States^34^ and 7% in Europe.^8^ It is now recognized as a clinical entity, regardless of its underlying cause.^4^ A variety of etiologically diverse disorders, such as diabetic neuropathy, a neurodegenerative, vascular, autoimmune condition, tumor, trauma, infection, toxicity exposure, or chemotherapy, could damage nerve function and increase pain sensation.^15,20^ According to epidemiological studies, many patients with NP do not receive appropriate pain treatment.^5,18^ Currently available medications for NP include opioids, nonsteroidal anti-inflammatory analgesics, antidepressants, anticonvulsants, antiarrhythmics, and others.^21^ However, NP requires a long-term treatment course, which will inevitably result in adverse drug reactions.^39^

Electroacupuncture (EA) is now widely used due to its superior efficacy, objectivity, and controllability. Recently, EA has been used to prevent and treat a variety of disorders; however, EA-induced antinociception has received the most attention because it could be applied as a complementary and alternative method of managing chronic pain with few side effects.^32,56^ Electroacupuncture has been reported to improve antinociception and reduce the minimum infusion rate of anesthetic agents such as alfaxalone (ALF)^30^ and propofol^31^ and minimize their side effects. The EA-induced antinociception is mediated by the release of endogenous opioids such as enkephalin, β-endorphin, and dynorphin in the central nervous system^12^ and activation of spinal opioidergic, α_2_ and β-adrenergic pathways.^13^ In addition, EA could relieve pain in a cute gout arthritic rats by locally upregulating β-endorphin expression.^11^ Moreover, EA could modulate the inhibitory neurotransmitter gamma-aminobutyric acid (GABA) and excitatory neurotransmitter glutamate in the spinal and higher brain regions in NP models.^25,56^ In addition, the efficacy of EA has also been substantiated in various experimental pain models, including osteoarthritis,^24^ inflammatory,^49^ and neuropathic^47^ models.

Gamma-aminobutyric acid is the main inhibitory neurotransmitter in the central nervous system.^10^ Diazepam-binding inhibitor (DBI) and its processing products are endogenous modulators of γ-aminobutyrate (GABAA). As a competitive binding inhibitor of diazepam, DBI binds to GABA receptors to produce negative allosteric modulation and inhibition of GABA-induced currents.^6^ Overexpression of DBI has been evidenced to inhibit GABA receptor signaling in vivo.^1^ Alfaxalone is a neuroactive steroid with potent anesthetic and antinociceptive properties by inhibitory actions on GABAA receptors. A recent study has proved that ALF could prevent hyperalgesia and anxiety-like behaviors in mice.^52^ Previous reports have shown that anesthetics administered at the subanesthetic levels could safely mitigate pain.^28,42^ Alfaxalone has several merits compared to other anesthetic agents, including a broad margin of safety, neuroprotection, and fewer accumulative effects after repeated doses^37,41^; however, the effect of subanesthetic ALF has not yet been studied. Hence, this study aims to investigate the potential role of EA and ALF as a novel target to alleviate spared nerve injury–induced NP. Our hypothesis was that EA, when combined with ALF, would synergistically mitigate NP through downregulating DBI, thereby enhancing the GABAergic effect.

## 2. Material and methods

### 2.1. Animals

Sixty healthy male C57BL/6 mice aging 6 to 8 weeks and weighing 18 to 22 g were enrolled in this study. Mice were purchased from the Experimental Animal Center of Huazhong Agricultural University, Wuhan, China, with a sale license number of SCXK (Hubei) 2020-0019. Mice were housed in a quiet environment with a temperature control of 22 ± 1°C, a humidity of 50% to 60%, a 12-hour light–dark cycle, and allowed free access to fresh water and food. One week before the start of the experimental manipulations, mice were acclimatized to the surrounding environment and experimental conditions. Before the experiment, mice were tested for normal pain threshold, and those who showed abnormal response timing and/or deviant behavior were not included in the experiment. All experimental procedures were conducted in compliance with the guidelines of the ethical committee of Huazhong Agricultural University (HZAUMO-2022-0211).

### 2.2. Experimental design

**Figure 1.**
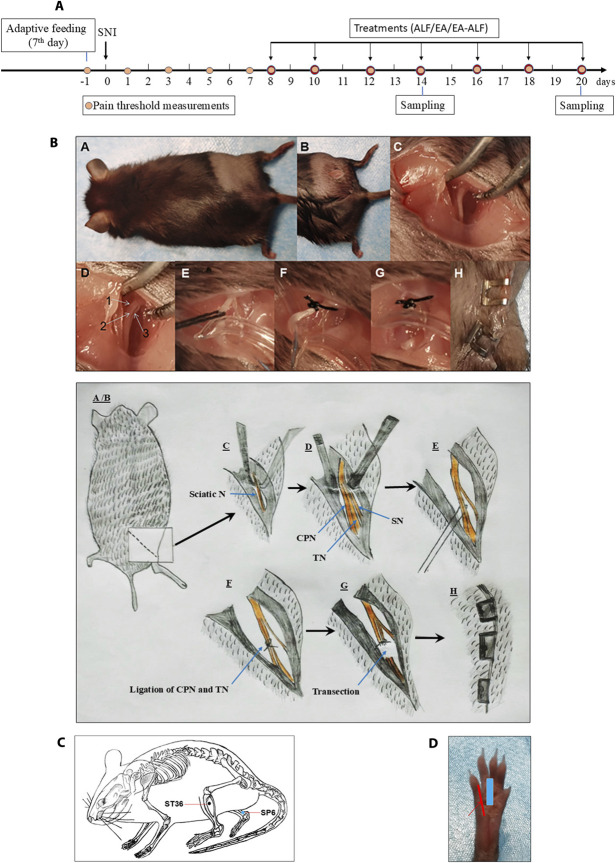
(a) Study timeline illustrating alfaxalone (ALF), electroacupuncture (EA), and EA-ALF treatment protocols of spared nerve injury (SNI) and spinal cord sampling in the SNI-ALF (n = 12), the SNI-EA (n = 12), and the SNI-EA-ALF (n = 12) groups. (b) Surgical procedures of SNI in mice with an illustrating diagram. (A) Mouse was placed in a prone position after anesthesia, and the surgical area was aseptically prepared. (B) A longitudinal incision was made in the middle of the lateral thigh. (C) The biceps femoris muscle (BFM) was exposed, and a careful blunt dissection was made through it to expose the trifurcation of the sciatic nerve. (D) Exposure of the sciatic nerve and its 3 branches: (1) common peroneal nerve (CPN), (2) tibial nerve (TN), and (3) sural nerve (SN). (E) A 5-0 silk suture was passed under CPN and TN. (F) Ligation of CPN and TN, leaving SN free. (G) Cutting of the ligated nerves and removal of a segment of 2 to 4 mm distal to the ligation. (H) Muscle was sutured, and the skin incision was closed. (c) Schematic diagram of the acupoint location of “Zusanli (ST 36) and Sanyinjiao (SP6)” in mice. (d) Schematic representation showing the position of the Von Frey probe for mechanical stimulation of the plantar aspect of the right hind paw in mice (red line). The central pad of the paw must be evaded (blue line).

After 7 days of adaptive feeding, a total of 60 mice were randomly allocated into 5 equal-sized groups using a computer program (www.randomizer.org) in this prospective, randomized, blinded experiment: (1) sham control (n = 12) group: the sciatic nerve and its branches (common peroneal, tibial, and sural) were surgically exposed but were neither ligated nor transected. (2) Spared nerve injury (SNI; n = 12) group: selective nerve injury was performed via ligating and transecting common peroneal and tibial branches of the sciatic nerve. (3) SNI-ALF group (n = 12): mice underwent SNI and were treated with ALF. (4) SNI-EA group (n = 12): mice underwent SNI and received EA. (5) SNI-EA-ALF group (n = 12): mice underwent SNI and received EA and ALF. For successful establishment of the SNI model before the start of the treatment protocols, pain thresholds were measured at baseline, 1, 3, 5, and 7 days postoperatively (PO). An investigator who was blinded to treatments measured mechanical and thermal pain thresholds in all mice. Mice in the 3 treatment groups (SNI-ALF, SNI-EA, and SNI-EA-ALF) were given the first treatment at 8 days PO and repeated once every 2 days for 7 treatments in total (Fig. 1A). The mechanical and thermal pain were tested after the first (8 days PO), second (10 days PO), third (12 days PO), fourth (14 days PO), fifth (16 days PO), sixth (18 days PO), and seventh (20 days PO) treatments. In the SNI-ALF group, ALF was injected alone intraperitoneally (IP) at a subanesthetic dose of 50 mg/kg, and the mice in the SNI-EA group received EA for 30 minutes. Although those in the SNI-EA-ALF group received EA for 30 minutes, followed by IP injection of ALF at a dose of 25 mg/kg. After the fourth (14 days PO) and seventh (20 days PO) treatments, 6 mice were randomly selected from each group and sacrificed for sample collection. Spinal cord samples (L4–L6) were harvested, weighed, and frozen in liquid nitrogen for western blotting and qPCR detection of pain biomarkers, including genes and protein levels of diazepam-binding inhibitor (DBI) and GABAA.

#### 2.2.1. Surgical induction of spared nerve injury model

The mice were anesthetized by injecting sodium pentobarbital (Nembutal 1%; 50 mg/kg; IP). Although under anesthesia, the mouse was placed on heating pads, then positioned and secured on the operating table in a prone position. The SNI model was conducted according to the method described by Cichon et al. 2018.^14^ Shortly, a 1- to 1.5-cm skin incision is made at the right middle thigh region, using the femur bone as a landmark, followed by a blunt dissection of the biceps femoris muscle (BFM) to expose the sciatic nerve and its 3 terminal branches: the sural, common peroneal, and tibial nerves. The common peroneal nerve (CPN) and the tibial nerve (TN) were tightly ligated with 5-0 silk, keeping the sural nerve (SN) intact. The ligated nerves were transected distal to the ligation, and a 2- to 4-mm section was removed to prevent nerve regeneration (Fig. 1B). Great care was taken during the operation to avoid damaging or stretching the spared sural nerve. In the sham group, the sciatic nerve branches were just exposed and not ligated or severed. The muscles and skin were then closed. Mice who showed self-mutilation or paralysis after the surgery were excluded from the experiment.

#### 2.2.2. Electroacupuncture stimulation

Electroacupuncture was performed at a predetermined time of day (9:00 am) as previously reported in other literature.^26,36^ Briefly, each mouse was gently placed into a specially designed polyethylene holder, with its hind legs and tail exposed. Before the experiment, mice were acclimated to the holder 30 minutes daily for 3 consecutive days at room temperature of 22 ± 1°C. The skin of the hind legs of the mice was sterilized with 75% alcohol. Stainless-steel needles (0.30 mm in diameter, 13 mm in length) were inserted into bilateral “Zusanli” acupoints (ST36, a notch 4 mm lateral to the anterior tuber point of the tibia, 2–3 mm depth) and “Sanyinjiao” acupoint (SP6, 3 mm proximal to the medial malleolus at the posterior border of the tibia, 2–3 mm depth) in the hind legs (Fig. 1C). The needles were connected with WQ-6F Electronic Acupuncto-scope (Beijing Xindonghua Electronic Instrument Co., Ltd., Beijing, China). In accordance with Zeng et al.,^54^ the EA stimuli were set as square waves, 2 Hz in frequency, with an amplitude of 3 mA, for 30 minutes.

#### 2.2.3. Nociceptive threshold measurement

##### 2.2.3.1. Mechanical sensitivity

Mechanical nociceptive threshold was determined by measuring the paw withdrawal threshold (PWT) of the right hind paws in response to mechanical stimulation. Before testing, the mice were acclimatized in individual plastic enclosures (12 × 22 × 18 cm^3^) on a metal mesh stand for 15 minutes. Paw withdrawal threshold was measured using an electronic Von Frey aesthesiometer (Beijing Zhongshi Dichuang Technology Development Co., Ltd., Beijing, China), which consists of a force transducer with a 0.5-mm diameter polypropylene rigid tip. The transducer was used to apply ascending pressure to the lateral plantar surface of the right hind paw, avoiding the mid-digital pad (Fig. 1D). The force causing the withdrawal response was automatically recorded. For each mouse, 3 measurements were taken with a 5-minute interval, and the average value of PWT was calculated.

##### 2.2.3.2. Thermal sensitivity

Thermal sensitivity was assessed by measuring paw withdrawal latency (PWL) with a hotplate analgesia meter, as previously described in another report.^33^ Before the measurement, the mice from each group were placed in the test room for 30 minutes to adapt to the environment. At 55°C, the mouse was placed on the hotplate's surface, and response was measured by recording the latency to lick, flutter, or splay hind paw (seconds) or an attempt to jump out of the apparatus, with a maximum cut-off time of 30 seconds. Once a response was detected or the cut-off time had passed, the mouse was removed from the hotplate and returned to its home cage. Between 2 measurements, the heated surface was wiped with 75% alcohol to avoid other factors, such as odor, which may affect the responses of other mice, and keep the temperature of the hotplate surface consistent.

#### 2.2.4. Quantitative Reverse Transcription Polymerase Chain Reaction

Total RNA of the spinal cord L4–6 was extracted using Trizol reagent (Invitrogen, Carlsbad, CA) and subsequently used for cDNA synthesis with a First Strand cDNA Synthesis Kit (TOYOBO, OSA, Japan). SYBR Premix Ex Taq II (Takara Bio Inc., Siga, Japan) was used for amplifying the GABA subtypes (A1, A2, A3, and A4) and DBI genes with the Step One Real-Time PCR System (Applied Biosystems, Carlsbad, CA). According to the nucleotide sequences of mouse GABA and DBI genes in the National Center of Biotechnology Information (NCBI), primers specific for GABA subtypes and DBI genes were designed and synthesized by Jinkairui Biological Engineering Co., Ltd, Hubei, Wuhan, China (Table 1). The mRNAs of GABAA and DBI genes relative to glyceraldehyde-3-phosphate dehydrogenase (GAPDH) were quantified with the 2^-∆Ct^ method, where (∆Ct = Ct_target gene_ − Ct_GAPDH_).

**Table 1:** Primer sequences of mouse glyceraldehyde-3-phosphate dehydrogenase (GAPDH), gamma-aminobutyric acid subtypes A (GABAA ), and diazepam-binding inhibitor genes.

Gene	Forward primer	Reverse primer
GAPDH	CAG​AAG​ACT​GTG​GAT​GGC​CC	ATC​CAC​GAC​GGA​CAC​ATT​GG
GABAA1	CGT​CAG​CAA​AAT​CGA​CCG​AC	CTA​TTG​ATG​GGG​TGT​GGG​GG
GABAA2	TGA​CTC​CGT​TCA​GGT​TGC​TC	TTC​TTG​CTG​GCA​CCG​ATT​CT
GABAA3	GAC​GAC​AAG​AAC​CTG​GGG​AC	TCT​GAC​ACA​GGG​CCA​AAA​CT
GABAA4	GGG​CCC​GTT​TCT​GAT​GTT​GA	ACT​CCG​CAC​TTA​TGG​TGA​GT
DBI	ACA​AGC​TAC​TGT​GGG​CGA​TG	TTG​TTC​CAC​GAG​TCC​CAC​TT

DBI, diazepam-binding inhibitor; GABAA1, γ-aminobutyrate A1 isoform receptor.

#### 2.2.5. Western blotting

The frozen spinal cord L4–6 was ground and mixed with cold RIPA Lysate Buffer according to the manufacturer's instructions (Beyotime Biotechnology Co., Ltd., Shanghai, China). Forty micrograms of proteins were subjected to 10% SDS-PAGE and transferred to a PVDF membrane using a minigel and mini transblot apparatus (Bio-Rad, Hercules, CA). After being incubated in 5% nonfat milk containing 0.1% Tween-20 for 2 hours, the membrane was infiltrated with the primary rabbit anti-γ-aminobutyrate A1 isoform receptor (GABAA1) (1:1000, Santa Cruz Biotechnology Co., Ltd., Shanghai, China), rabbit anti-DBI (1:1000, Abcam, Cambridge Biomedical Campus, Cambridge, United Kingdom), and mouse α-tubulin antibodies (1:1000, Wuhan Sanying Biotechnology Co., Ltd, Wuhan, China) at 4°C overnight. The membrane was washed in Tris-Buffered saline Tween-20 (TBST) 3 times and incubated in the corresponding horseradish peroxidase (HRP)-conjugated secondary antibody (1:3000, Servicebio, Wuhan, China) at room temperature for 1 hour. Alpha-tubulin was used as an internal control. The antigen–antibody complex was reacted with HRP substrate (Millipore, Burlington, MA) and visualized using the ImageQuant LAS 4000 minutes CCD camera (GE Healthcare, Boston, MA). The bands were analyzed by Quantity One software (Bio-Rad, Hercules, CA). Values of GABAA1 and DBI were represented as the optical density ratio of the target protein bands to the related α-tubulin bands.

#### 2.2.6. Statistical analysis

All experimental data were expressed as mean ± standard deviation (SD), and the statistical analysis of the data was performed using GraphPad Prism 7.0 software (GraphPad Software, Inc., San Diego, CA). Two-way analysis of variance (ANOVA) was used to compare variables between groups with Tukey multiple comparison test. The Kolmogorov–Smirnov test was used to confirm the normal distribution of data. The differences were considered significant at *P* < 0.05.

## 3. Results

### 3.1. Validation of spared nerve injury neuropathic pain model

**Figure 2.**
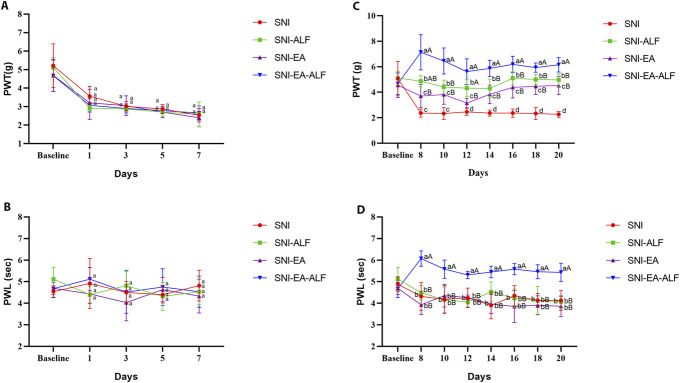
(A and B) Paw withdrawal threshold (PWT; g) and paw withdrawal latency (PWL; sec) of the spared nerve injury (SNI) model at baseline, days 1, 3, 5, and 7 postoperatively (PO) in the SNI (n = 12), SNI-ALF (n = 12), SNI-EA (n = 12), and SNI-EA-ALF (n = 12) groups. (C and D) Effect of alfaxalone (ALF), electroacupuncture (EA), and EA-ALF treatment protocols on PWT and PWL after the first, second, third, fourth, fifth, sixth, and seventh treatments (8, 10, 12, 14, 16, 18, and 20 days PO, respectively) in the SNI-ALF (n = 12), SNI-EA (n = 12), and SNI-EA-ALF (n = 12) groups. (a–d) Values with different lowercase letters indicate significant differences between each treatment group and the SNI group at the same time point (*P* < 0.05). (A and B) Values with different uppercase letters indicate significant differences between treatment groups at the same time point (*P* < 0.05).

Before the start of the treatment regimens of SNI, we validated the SNI neuropathic pain model by measuring mechanical and thermal pain sensitivities 1 week after the induction surgery in all operated mice. As shown in Figure 2A, the operated mice in the SNI, SNI-ALF, SNI-EA, and SNI-EA-ALF groups displayed a significant decrease in the PWT compared to the baseline at days 1, 3, 5, and 7 PO (*P* < 0.05). However, nonsignificant fluctuations in the PWL were noted in all groups compared to the baseline (Fig. 2B). In this current study, the SNI mice showed no signs of weight loss or self-mutilation. Only a few mice had lame or slightly valgus right hind limbs on day 1 PO, but they all recovered quickly. The social interactions and play behaviors of the SNI mice were nearly identical to those of the sham mice.

### 3.2. Effect of electroacupuncture combined with alfaxalone on mechanical and thermal pain sensitivities in neuropathic mice

#### 3.2.1. Mechanical pain sensitivity

Mice in the SNI-ALF, SNI-EA, and SNI-EA-ALF groups showed a significant increase in the values of PWT compared to the SNI group after the third (12 days PO) (*P* = 0.0060, *P* = 0.0036, *P* = 0.0034, respectively), fourth (14 days PO) (*P* = 0.0026, *P* = 0.0268, *P* = 0.0001, respectively), fifth (16 days PO) (*P* = 0.0009, *P* = 0.0053, *P* = 0.0001, respectively), sixth (18 days PO) (*P* = 0.0005, *P* = 0.0014, *P* = 0.0003, respectively), and seventh (20 days PO) (*P* < 0.0001, *P* = 0.0012, *P* < 0.0001, respectively) treatments. When comparing the treatment groups, the SNI-EA-ALF group demonstrated a remarkable rise in the PWT compared to the SNI-ALF and SNI-EA groups after the fourth (14 days PO) (*P* = 0.0298, *P* = 0.0254, respectively), fifth (16 days PO) (*P* = 0.0175, *P* = 0.0475, respectively), sixth (18 days PO) (*P* = 0.0379, *P* = 0.0491, respectively), and seventh (20 days PO) (*P* = 0.0181, *P* = 0.0467, respectively) treatments (Fig. 2C).

#### 3.2.2. Thermal pain sensitivity

The SNI-ALF and SNI-EA groups showed nonsignificant differences in the PWL compared to the SNI group; however, a significant increase in the PWL was observed in the SNI-EA-ALF group as compared to the SNI group after the first (8 days PO) (*P* = 0.0100), second (10 days PO) (*P* = 0.0003), third (12 days PO) (*P* = 0.0183), fourth (14 days PO) (*P* = 0.0208), fifth (16 days PO) (*P* = 0.0042), sixth (18 days PO) (*P* = 0.0020), and seventh (20 days PO) (*P* = 0.0262) treatments. Furthermore, mice in the SNI-EA-ALF group displayed a significant increase in the PWL values compared to those in the SNI-ALF and SNI-EA groups after the first (8 days PO) (*P* = 0.0020, *P* = 0.0027, respectively), second (10 days PO) (*P* = 0.0120, *P* = 0.0013, respectively), third (12 days PO) (*P* < 0.0001, *P* = 0.0001, respectively), fourth (14 days PO) (*P* = 0.0362, *P* = 0.0003, respectively), fifth (16 days PO) (*P* = 0.0085, *P* = 0.0026, respectively), sixth (18 days PO) (*P* = 0.0344, *P* = 0.0034, respectively), and seventh (20 days PO) (*P* = 0.0030, *P* < 0.0001, respectively) treatments (Fig. 2D).

### 3.3. Effect of electroacupuncture combined with alfaxalone on γ-aminobutyrate and diazepam-binding inhibitor mRNA expression level

#### 3.3.1. γ-Aminobutyrate mRNA

**Figure 3.**
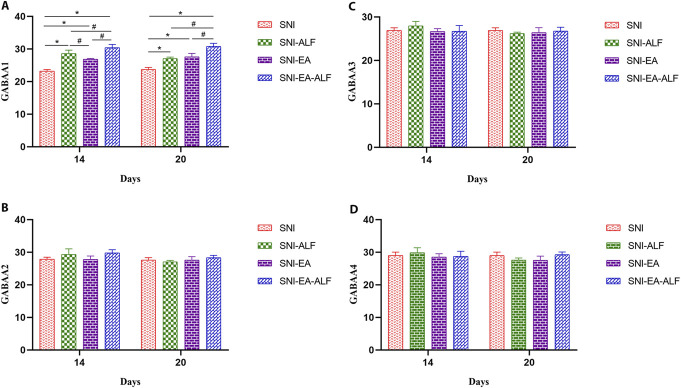
Effect of alfaxalone (ALF), electroacupuncture (EA), and EA-ALF treatment protocols on the mRNA expression levels of gamma-aminobutyric acid type A1 (GABAA1) (A), GABAA2 (B), GABAA3 (C), and GABAA4 (D). The level of mRNA after the fourth and seventh treatments (14 days and 20 days postoperatively, respectively) in the spared nerve injury (SNI) (n = 12), SNI-ALF (n = 12), SNI-EA (n = 12), and SNI-EA-ALF (n = 12) groups. *Significant differences between each treatment group and the SNI group (*P* < 0.05). #Significant differences between the treatment groups (*P* < 0.05).

Mice in the SNI-ALF, SNI-EA, and SNI-EA-ALF groups exhibited a significant increase in the levels of GABAA1 mRNA expression compared to those in the SNI group after the fourth (14 days PO) (*P* = 0.0003, *P* < 0.0001, *P* < 0.0001, respectively) and seventh (20 days PO) (*P* = 0.0020, *P* = 0.0003, *P* = 0.0002, respectively) treatments. Compared to the SNI-ALF and SNI-EA groups, the SNI-EA-ALF group showed a significant increase in the GABAA1 expression after the fourth (14 days PO) (*P* = 0.0202, *P* = 0.0008, respectively) and seventh (20 days PO) (*P* = 0.0012, *P* = 0.0361, respectively) treatments (Fig. 3A). The expression levels of GABAA2, GABAA3, and GABAA4 mRNA in the SNI-ALF, SNI-EA, and SNI-EA-ALF groups had no significant changes compared to the SNI group (Figs. 3B–D).

#### 3.3.2. Diazepam-binding inhibitor mRNA

**Figure 4.**
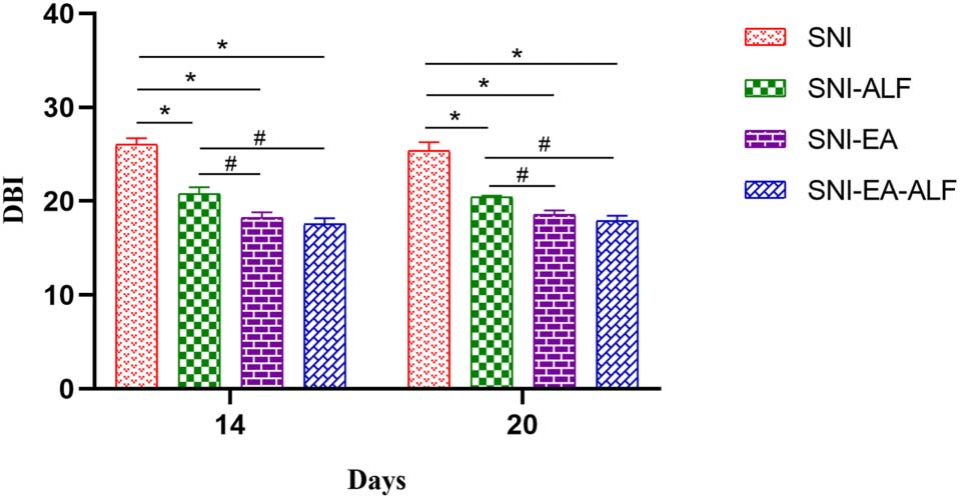
Effect of alfaxalone (ALF), electroacupuncture (EA), and EA-ALF treatment protocols on the expression levels of diazepam binding inhibitor (DBI) mRNA after the fourth and seventh treatments (14 days and 20 days postoperatively, respectively) in the spared nerve injury (SNI) (n = 12), SNI-ALF (n = 12), SNI-EA (n = 12), and SNI-EA-ALF (n = 12) groups. *Significant differences between each treatment group and the SNI group (*P* < 0.05). #Significant differences between the treatment groups (*P* < 0.05).

The expression level of DBI mRNA was significantly decreased in the SNI-ALF, SNI-EA, and SNI-EA-ALF groups compared to that in the SNI group after the fourth (14 days PO) (*P* < 0.0001, *P* = 0.0017, *P* < 0.0001, respectively) and seventh (20 days PO) (*P* = 0.0308, *P* < 0.0001, *P* < 0.0001, respectively) treatments. The SNI-EA-ALF group showed a significant downregulation in the DBI mRNA expression compared to the SNI-ALF after the fourth (14 days PO) (*P* = 0.0005) and seventh (20 days PO) (*P* = 0.0086) treatments (Fig. 4).

### 3.4. Effect of electroacupuncture combined with alfaxalone on γ-aminobutyrate and diazepam-binding inhibitor protein expression level

#### 3.4.1. γ-Aminobutyrate A1 isoform receptor protein

**Figure 5.**
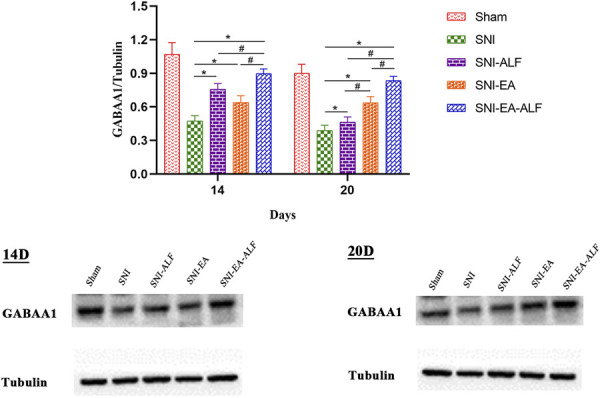
Effect of alfaxalone (ALF), electroacupuncture (EA), and EA-ALF treatment protocols on GABAA1 protein expression after the fourth and seventh treatments (14 days and 20 days postoperatively, respectively) in the sham (n = 12), spared nerve injury (SNI) (n = 12), SNI-ALF (n = 12), SNI-EA (n = 12), and SNI-EA-ALF (n = 12) groups. *Significant differences between each treatment group and the SNI group (*P* < 0.05). #Significant differences between the treatment groups (*P* < 0.05). GABAA1, γ-aminobutyrate A1 isoform receptor.

To further verify the effect of EA and ALF on the expression of GABA, western blotting was used to detect the protein expression of GABAA1. In comparison to the SNI group, the expression of GABAA1 protein was significantly increased in the SNI-ALF, SNI-EA, and SNI-EA-ALF groups after the fourth (14 days PO) (*P* = 0.0036, *P* = 0.0125, *P* = 0.0001, respectively) and seventh (20 days PO) (*P* = 0.0203, *P* = 0.0019, *P* < 0.0001, respectively) treatments. The SNI-EA-ALF group showed a significant upregulation in the GABAA1 protein compared to that observed in the SNI-ALF and SNI-EA groups after the fourth (14 days PO) (*P* = 0.0100, *P* = 0.0040, respectively) and seventh (20 days PO) (*P* < 0.0001, *P* = 0.0015, respectively) treatments (Fig. 5).

#### 3.4.2. Diazepam-binding inhibitor protein

**Figure 6.**
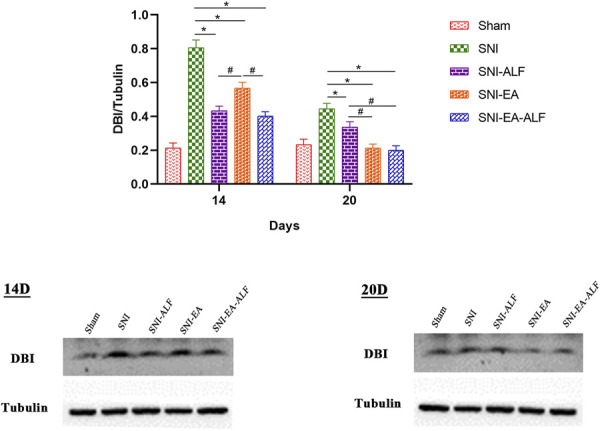
Effect of alfaxalone (ALF), electroacupuncture (EA), and EA-ALF treatment protocols on diazepam binding inhibitor (DBI) protein expression after the fourth and seventh treatments (14 days and 20 days postoperatively, respectively) in the sham (n = 12), spared nerve injury (SNI) (n = 12), SNI-ALF (n = 12), SNI-EA (n = 12), and SNI-EA-ALF (n = 12) groups. *Significant differences between each treatment group and the SNI group (*P* < 0.05). #Significant differences between the treatment groups (*P* < 0.05).

There were significant decreases in the expression of DBI protein in the SNI-ALF, SNI-EA, and SNI-EA-ALF groups compared to the SNI group after the fourth (14 days PO) (*P* < 0.0001, *P* = 0.0017, and *P* < 0.0001, respectively) and seventh (20 days PO) (*P* = 0.0308, *P* < 0.0001, *P* < 0.0001, respectively). The DBI protein expression was significantly downregulated in the SNI-EA-ALF group compared to the SNI-EA group after the fourth (14 days PO) (*P* = 0.0005) treatment and the SNI-ALF group after the seventh (20 days PO) (*P* = 0.0086) treatment (Fig. 6).

#### 3.4.3. Comparison of γ-aminobutyrate A1 isoform receptor and diazepam-binding inhibitor protein expression between fourth and seventh treatments

**Figure 7.**
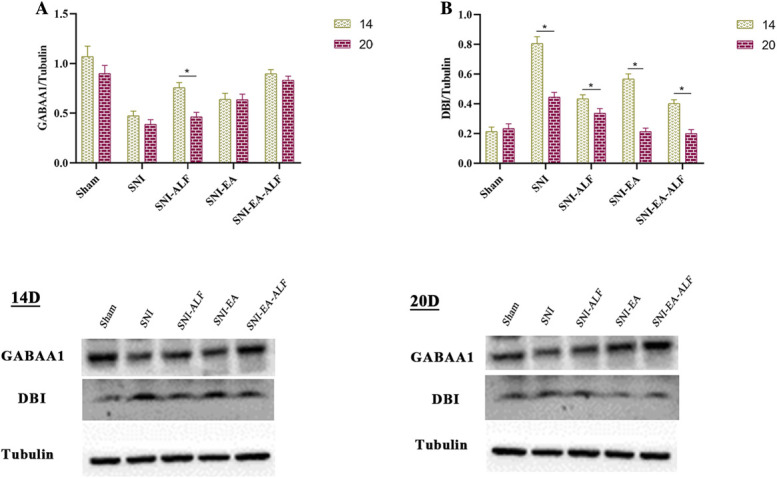
Expression of gamma-aminobutyric acid type A1 (GABAA1) and diazepam binding inhibitor (DBI) protein between the fourth and seventh treatments (14 days and 20 days postoperatively, respectively) in the sham (n = 12), spared nerve injury (SNI) (n = 12), SNI-ALF (n = 12), SNI-EA (n = 12), and SNI-EA-ALF (n = 12) groups. (A) Comparison of GABAA1 protein expression between the fourth and seventh treatments in the same group. (B) Comparison of DBI protein expression between the fourth and seventh treatments in the same group. *Significant differences between the treatments in the same group (*P* < 0.05). ALF, alfaxalone; EA, electroacupuncture.

When comparing the protein expression between the fourth (14 days PO) and seventh (20 days PO) treatments within each group, the GABAA1 protein expression after the seventh (20 days PO) in the SNI-ALF group was significantly lower than that after the fourth (14 days PO) treatment (*P* < 0.0001). However, there were nonsignificant alterations in the GABAA1 expression in the SNI-EA group and the SNI-EA-ALF group. Concerning DBI protein expression, the SNI, SNI-ALF, SNI-EA, and SNI-EA-ALF groups showed a significant reduction after the seventh (20 days PO) treatment compared to the fourth (14 days PO) treatment (*P* < 0.0001, *P* = 0.0010, *P* < 0.0001, *P* < 0.0001, respectively) (Fig. 7).

## 4. Discussion

The prevalence of NP has been rapidly increasing in the general population around the world, and its remedy is becoming more challenging. A variety of pharmacotherapies have been used to treat pain in patients with NP, but they are ineffective and come with substantial adverse effects, including vascular and cardiac disorders, peripheral edema, pain, vertigo, blurred vision, and weight gain.^2,3^ Therefore, this current study has been planned to investigate the combination of EA and ALF as a novel therapeutic protocol for mitigating NP. To investigate the efficacy of the treatment regime, it is pivotal to choose a successful experimental model mimicking the hallmark symptoms observed in patients with NP. Several NP models had been proposed in the previous literature, including SNI,^14^ spinal nerve ligation (SNL),^53^ partial sciatic nerve ligation (PNL),^38^ and sciatic nerve chronic compression injury (CCI).^35^ In this current study, the SNI model has been advocated because it is considered simpler, easier to manipulate, and produces earlier (24 hours) and longer (>6 months) NP-like behaviors as compared to other models.^16,16,43^ As well, we validated the NP model 1 week (pretest period) before the commencement of treatments. Our model was more sensitive to mechanical but not to 55°C thermal stimulation, which is in line with previous SNI-induced NP models.^9,14,16^ Pain sensitization was maintained throughout the pretest period, indicating that the NP model was well established. In this current study, SNI mice treated with EA plus ALF was associated with a significant increase in nociceptive thresholds as compared to the use of EA or ALF alone. Electroacupuncture was found to attenuate nociceptive behaviors in SNI rats through suppressing synaptotagmin-1 (Syt-1) protein expression in the spinal cord.^48^ In addition, EA was evidenced to diminish P2X7 receptor expression that is involved in the improvement of neurobehavioral performance in SNL rats.^50^ As well, ALF has been reported to inhibit stress-induced hyperalgesia and anxiety sensitivity in mice.^52^ Based on previous literature, our findings suggest that EA and ALF could act synergically to improve antinociceptive outcomes in NP.

Neuropathic pain frequently causes a disruption in the nociceptive neurotransmission, which encompasses the inhibitory (mostly GABAergic) and excitatory (mostly glutamatergic) systems.^22,27^ Once the GABAA receptor is activated, the ligand-gated chloride channel opens and chloride influxes within the postsynaptic neurons, causing hyperpolarization and the blockage of neural impulse transmission. A potentiation of GABAA-mediated chloride influx has been shown to have a remarkable antinociceptive outcome in a variety of NP models.^17,55^

Electroacupuncture is one of the most effective and safe methods for treating pathological pain caused by nerve damage, but the mechanisms by which EA could modulate NP signaling are not widely understood.^29^ Both clinical and experimental studies have shown that endogenous opiate peptides are involved in the EA-induced antinociception. According to animal experiments, the release of endogenous neuropeptides depends on the EA frequency of EA. The 2 Hz frequency accelerates the release of enkephalin, dynorphin, and endorphins, whereas the 100-Hz frequency selectively increases the release of morphine.^23^ Furthermore, the low-frequency EA could trigger long-lasting antinociception more than the high-frequency EA.^45,51^ A recent study has also reported that GABAA γ2 gene expression in the spinal cord contributes to the EA antinociception in SNL rats.^56^ Electroacupuncture has been reported to enhance antinociception as well as reduce the dose of antinociceptive drugs.^30,31^ Furthermore, EA could reduce the demand of opiate in patients with chronic pain.^55^

Alfaxalone at a dose range of 80 to 120 mg/kg IP has been reported to induce anesthesia in mice.^19,46^ In this current study, subanesthetic ALF at a dose rate of 50 mg/kg IP was tested and chosen. The EA-ALF combination resulted in a significant rise in GABAA expression, reducing the dosage requirements of ALF when used alone, thereby achieving the concept of multimodal therapy of NP.^40^ As a consequence of peripheral neuropathy, DBI has been revealed in the spinal cord, resulting in the development of acute symptoms of NP by triggering the excitatory postsynaptic potential.^7,44^ As well, Alfonso et al. reported that DBI expression could counteract the inhibitory effect of GABA signaling on the subventricular zone in the cerebral cortex.^1^ In this current study, the use of only male mice may have limited the value of observation as a true representation of both sexes. It is becoming standard to test mice of both sexes to capture potential sex differences. However, our study provided valuable data and revealed that the EA-ALF combination could improve the expression of GABAA receptor via downregulating DBI in the spinal cord and potentiate the analgesic and antineuropathic value of the treatment, representing a promising therapeutic target for NP. Also, our findings might encourage future clinical research to use the EA plus ALF as an essential multimodal regimen for NP.

## Disclosures

The authors have no conflict of interest to declare.

## References

[1] Alfonso J Le Magueresse C Zuccotti A Khodosevich K Monyer H. Diazepam binding inhibitor promotes progenitor proliferation in the postnatal SVZ by reducing GABA signaling. Cell Stem Cell 2012;10:76–87. 22226357 10.1016/j.stem.2011.11.011

[2] Arneric SP Laird JMA Chappell AS Kennedy JD. Tailoring chronic pain treatments for the elderly: are we prepared for the challenge? Drug Discov Today 2014;19:8–17. 24001595 10.1016/j.drudis.2013.08.017

[3] Attal N Bouhassira D. Pharmacotherapy of neuropathic pain: which drugs, which treatment algorithms? PAIN 2015;156(suppl 1):S104–14. 25789426 10.1097/01.j.pain.0000460358.01998.15

[4] Attal N Fermanian C Fermanian J Lanteri-Minet M Alchaar H Bouhassira D. Neuropathic pain: are there distinct subtypes depending on the aetiology or anatomical lesion? PAIN 2008;138:343–53. 18289791 10.1016/j.pain.2008.01.006

[5] Attal N Lanteri-Minet M Laurent B Fermanian J Bouhassira D. The specific disease burden of neuropathic pain: results of a French nationwide survey. PAIN 2011;152:2836–43. 22019149 10.1016/j.pain.2011.09.014

[6] Borchardt JS Blecker LM Satyshur KA Czajkowski C. The diazepam binding inhibitor’s modulation of the GABA-A receptor is subunit-dependent. bioRxiv. 2021. doi:10.1101/2021.08.13.456294.

[7] Bormann J. Electrophysiological characterization of diazepam binding inhibitor (DBI) on GABAA receptors. Neuropharmacology 1991;30:1387–9. 1723508 10.1016/s0028-3908(11)80006-7

[8] Bouhassira D Lantéri-Minet M Attal N Laurent B Touboul C. Prevalence of chronic pain with neuropathic characteristics in the general population. PAIN 2008;136:380–7. 17888574 10.1016/j.pain.2007.08.013

[9] Bourquin A-F Süveges M Pertin M Gilliard N Sardy S Davison AC Spahn DR Decosterd I. Assessment and analysis of mechanical allodynia-like behavior induced by spared nerve injury (SNI) in the mouse. PAIN 2006;122:14.e1. 10.1016/j.pain.2005.10.036 16542774

[10] Brohan J Goudra BG. The role of GABA receptor agonists in anesthesia and sedation. CNS Drugs 2017;31:845–56. 29039138 10.1007/s40263-017-0463-7

[11] Chai W Tai Y Shao X Liang Y Zheng G Wang P Fang J Liu B. Electroacupuncture alleviates pain responses and inflammation in a rat model of acute gout arthritis. Evid Based Complement Altern Med. 2018;2018. doi:10.1155/2018/2598975 PMC5884439 29743920

[12] Cheng L-L Ding M-X Xiong C Zhou M-Y Qiu Z-Y Wang Q. Effects of electroacupuncture of different frequencies on the release profile of endogenous opioid peptides in the central nerve system of goats. Evid Based Complement Altern Med 2012;2012:476457. 10.1155/2012/476457 PMC3486625 23133494

[13] Choi J-W Kang S-Y Choi J-G Kang D-W Kim S-J Lee SD Park JB Ryu Y-H Kim H-W. Analgesic effect of electroacupuncture on paclitaxel-induced neuropathic pain via spinal opioidergic and adrenergic mechanisms in mice. Am J Chin Med 2015;43:57–70. 25640847 10.1142/S0192415X15500044

[14] Cichon J Sun L Yang G. Spared nerve injury model of neuropathic pain in mice. Bio Protocol 2018;8:e2777. 10.21769/BioProtoc.2777 PMC5906065 29682596

[15] Colloca L Ludman T Bouhassira D Baron R Dickenson AH Yarnitsky D Freeman R Truini A Attal N Finnerup NB. Neuropathic pain. Nat Rev Dis Prim 2017;3:1–19. 10.1038/nrdp.2017.2 PMC5371025 28205574

[16] Decosterd I Woolf CJ. Spared nerve injury: an animal model of persistent peripheral neuropathic pain. PAIN 2000;87:149–58. 10924808 10.1016/S0304-3959(00)00276-1

[17] Désarmenien M Feltz P Occhipinti G Santangelo F Schlichter R. Coexistence of GABAA and GABAB receptors on A delta and C primary afferents. Br J Pharmacol 1984;81:327–33. 6322896 10.1111/j.1476-5381.1984.tb10082.x PMC1986884

[18] Dworkin RH Panarites CJ Armstrong EP Malone DC Pham SV. Is treatment of postherpetic neuralgia in the community consistent with evidence-based recommendations? PAIN 2012;153:869–75. 22356792 10.1016/j.pain.2012.01.015

[19] Erickson RL Blevins CE Souza Dyer CDe Marx JO. Alfaxalone–xylazine anesthesia in laboratory mice (Mus musculus). J Am Assoc Lab Anim Sci 2019;58:30–9. 30572981 10.30802/AALAS-JAALAS-18-000010 PMC6351053

[20] Farquhar-Smith P. Chemotherapy-induced neuropathic pain. Curr Opin Support Palliat Care 2011;5:1–7. 21192267 10.1097/SPC.0b013e328342f9cc

[21] Finnerup NB Attal N Haroutounian S McNicol E Baron R Dworkin RH Gilron I Haanpää M Hansson P Jensen TS Kamerman PR Lund K Moore A Raja SN Rice ASC Rowbotham M Sena E Siddall P Smith BH Wallace M. Pharmacotherapy for neuropathic pain in adults: a systematic review and meta-analysis. Lancet Neurol 2015;14:162–73. 25575710 10.1016/S1474-4422(14)70251-0 PMC4493167

[22] Guo D Hu J. Spinal presynaptic inhibition in pain control. Neuroscience 2014;283:95–106. 25255936 10.1016/j.neuroscience.2014.09.032

[23] Han J-S. Acupuncture and endorphins. Neurosci Lett 2004;361:258–61. 15135942 10.1016/j.neulet.2003.12.019

[24] Huang J Zhuo L-S Wang Y-Y Peng Z-L Huang Y-R Wang Y Yang L. Effects of electroacupuncture on synovia IL-1beta and TNF-alpha contents in the rabbit with knee osteoarthritis. Zhen Ci Yan Jiu 2007;32:115–8. 17650656

[25] Huang C-P Lin Y-W Lee D-Y Hsieh C-L. Electroacupuncture relieves CCI-induced neuropathic pain involving excitatory and inhibitory neurotransmitters. Evid Based Complement Altern Med 2019;2019:6784735. 10.1155/2019/6784735 PMC6854981 31772598

[26] Koo S-T Kim S-K Kim E-H Kim J-H Youn D-H Lee B-H Chae Y-B Choi I-H Choi S-M. Acupuncture point locations for experimental animal studies in rats and mice. Korean J Acupunct 2010;27:67–78.

[27] Lee MC Nam TS Jung SJ Gwak YS Leem JW. Modulation of spinal GABAergic inhibition and mechanical hypersensitivity following chronic compression of dorsal root ganglion in the rat. Neural Plast 2015;2015:924728. 26451259 10.1155/2015/924728 PMC4584224

[28] Leung SYD Meng F Liu J Liu AJ Ng HLL Cheung CW Sau Ching Wong S. Sub-anaesthetic dose of propofol attenuates mechanical allodynia in chronic post-ischaemic pain via regulation of PTEN/PI3K/IL-6 signalling. Mol Pain 2023;19:17448069231185232. 37314769 10.1177/17448069231185232 PMC10293517

[29] Leung L. Neurophysiological basis of acupuncture-induced analgesia—an updated review. J Acupunct Meridian Stud 2012;5:261–70. 23265077 10.1016/j.jams.2012.07.017

[30] Liu L Abouelfetouh MM Salah E Sun R Nan S Ding M Ding Y. Determination of the minimum infusion rate of alfaxalone combined with electroacupuncture in goats. Animals 2021;11:2989. 34680008 10.3390/ani11102989 PMC8532749

[31] Liu L Abouelfetouh MM Sun R Salah E Nan S Ding M Song Y Ding Y. Determination of minimum infusion rate of propofol in combination with electroacupuncture in goats. Small Ruminant Res 2023;224:106979.

[32] Lv Z Shen L Zhu B Zhang Z Ma C Huang G Yin J Yu L Yu S Ding M Li J Yuan XC He W Jing XH Li M. Effects of intensity of electroacupuncture on chronic pain in patients with knee osteoarthritis: a randomized controlled trial. Arthritis Res Ther 2019;21:120. 31088511 10.1186/s13075-019-1899-6 PMC6518678

[33] Manglik A Lin H Aryal DK McCorvy JD Dengler D Corder G Levit A Kling RC Bernat V Hübner H Huang XP Sassano MF Giguère PM Löber S Da Duan Scherrer G Kobilka BK Gmeiner P Roth BL Shoichet BK. Structure-based discovery of opioid analgesics with reduced side effects. Nature 2016;537:185–90. 27533032 10.1038/nature19112 PMC5161585

[34] Mbrah AK Nunes AP Hume AL Zhao D Jesdale BM Bova C Lapane KL. Prevalence and treatment of neuropathic pain diagnoses among US nursing home residents. PAIN 2022;163:1370–7. 34711763 10.1097/j.pain.0000000000002525 PMC11519976

[35] Medeiros P Dos Santos IR Júnior IM Palazzo E da Silva JA Machado HR Ferreira SH Maione S Coimbra NC de Freitas RL. An adapted chronic constriction injury of the sciatic nerve produces sensory, affective, and cognitive impairments: a peripheral mononeuropathy model for the study of comorbid neuropsychiatric disorders associated with neuropathic pain in rats. Pain Med 2021;22:338–51. 32875331 10.1093/pm/pnaa206

[36] Minfeng R Jisheng H. Rat tail flick acupuncture analgesia model. Chin Med J (Engl) 1979;92:576–82. 113188

[37] Nadeson R Goodchild CS. Antinociceptive properties of neurosteroids II.: experiments with saffan® and its components alphaxalone and alphadolone to reveal separation of anaesthetic and antinociceptive effects and the involvement of spinal cord GABAA receptors. PAIN 2000;88:31–9. 11098097 10.1016/S0304-3959(00)00300-6

[38] Nicol LSC Dawes JM La Russa F Didangelos A Clark AK Gentry C Grist J Davies JB Malcangio M McMahon SB. The role of G-protein receptor 84 in experimental neuropathic pain. J Neurosci 2015;35:8959–69. 26063927 10.1523/JNEUROSCI.3558-14.2015 PMC4461694

[39] Noori SA Aiyer R Yu J White RS Mehta N Gulati A. Nonopioid versus opioid agents for chronic neuropathic pain, rheumatoid arthritis pain, cancer pain and low back pain. Pain Manag 2019;9:205–16. 30681031 10.2217/pmt-2018-0052

[40] Serrano Afonso A Carnaval T Videla Cés S. Combination therapy for neuropathic pain: a review of recent evidence. J Clin Med 2021;10:3533. 34441829 10.3390/jcm10163533 PMC8396869

[41] Serrao JM Goodchild CS. Alfaxalone anaesthesia increases brain derived neurotrophic factor levels and preserves postoperative cognition by activating pregnane-X receptors: an in vitro study and a double blind randomised controlled trial. BMC Anesthesiol 2022;22:401. 36564723 10.1186/s12871-022-01940-x PMC9789577

[42] Sheehy KA Lippold C Rice AL Nobrega R Finkel JC Quezado ZMN. Subanesthetic ketamine for pain management in hospitalized children, adolescents, and young adults: a single-center cohort study. J Pain Res 2017;10:787–95. 28435316 10.2147/JPR.S131156 PMC5388303

[43] Shields SD Eckert WA III Basbaum AI. Spared nerve injury model of neuropathic pain in the mouse: a behavioral and anatomic analysis. J Pain 2003;4:465–70. 14622667 10.1067/s1526-5900(03)00781-8

[44] Siiskonen H Oikari S Korhonen V-P Pitkänen A Voikar V Kettunen M Hakumäki J Wahlfors T Pussinen R Penttonen M Kiehne K Kaasinen SK Alhonen L Jänne J Herzig KH. Diazepam binding inhibitor overexpression in mice causes hydrocephalus, decreases plasticity in excitatory synapses and impairs hippocampus-dependent learning. Mol Cell Neurosci 2007;34:199–208. 17150371 10.1016/j.mcn.2006.10.013

[45] Silva ML Silva JRT Prado WA. The integrity of the anterior pretectal nucleus and dorsolateral funiculus is necessary for electroacupuncture-induced analgesia in the rat tail-flick test. Eur J Pain 2010;14:249–54. 19560380 10.1016/j.ejpain.2009.05.007

[46] Siriarchavatana P Ayers JD Kendall LV. Anesthetic activity of alfaxalone compared with ketamine in mice. J Am Assoc Lab Anim Sci 2016;55:426–30. 27423149 PMC4943613

[47] Sun T Cui CB Luo JG Zhang L Fu ZJ Song WG. Effect of electroacupuncture on the expression of spinal glial fibrillary acidic protein, tumor necrosis factor-alpha and interleukin-1beta in chronic neuropathic pain rats. Zhen Ci Yan Jiu 2010;35:12–6. 20458900

[48] Wan J Nan S Liu J Ding M Zhu H Suo C Wang Z Hu M Wang D Ding Y. Synaptotagmin 1 is involved in neuropathic pain and electroacupuncture-mediated analgesic effect. Int J Mol Sci 2020;21:968. 32024024 10.3390/ijms21030968 PMC7037106

[49] Wang WJ Lu J Niu CS Huang YR Ma Q Yg A Hao HW Li LM Tu Y. Effects of electroacupuncture of unilateral and bilateral “zusanli”(ST 36) on serum TNF-alpha, IL-1 and IL-4 levels in rats with chronic inflammatory pain. Zhen Ci Yan Jiu 2010;35:429–32. 21375016

[50] Wu Q Yue J Lin L Yu X Zhou Y Ying X Chen X Tu W Lou X Yang G Zhou K Jiang S. Electroacupuncture may alleviate neuropathic pain via suppressing P2X7R expression. Mol Pain 2021;17:1744806921997654. 33626989 10.1177/1744806921997654 PMC7934063

[51] Xing G-G Liu F-Y Qu X-X Han J-S Wan Y. Long-term synaptic plasticity in the spinal dorsal horn and its modulation by electroacupuncture in rats with neuropathic pain. Exp Neurol 2007;208:323–32. 17936754 10.1016/j.expneurol.2007.09.004

[52] Yoshizawa K Ukai S Kuroda J Yamauchi T Yamada D Saitoh A Iriyama S Nishino S Miyazaki S. Alfaxalone improved in acute stress‐induced tactile hypersensitivity and anxiety‐like behavior in mice. Neuropsychopharmacol Rep 2022;42:213–7. 35118831 10.1002/npr2.12233 PMC9216362

[53] Yowtak J Lee KY Kim HY Wang J Kim HK Chung K Chung JM. Reactive oxygen species contribute to neuropathic pain by reducing spinal GABA release. PAIN 2011;152:844–52. 21296500 10.1016/j.pain.2010.12.034 PMC3108328

[54] Zeng J Cui L Feng Y Ding M. Electroacupuncture relieves neuropathic pain via upregulation of glutamate transporters in the spinal cord of rats. Neurosci Lett 2016;620:38–42. 27026488 10.1016/j.neulet.2016.03.041

[55] Zheng Z Guo RJ Helme RD Muir A Da Costa C Xue CCL. The effect of electroacupuncture on opioid-like medication consumption by chronic pain patients: a pilot randomized controlled clinical trial. Eur J Pain 2008;12:671–6. 18035566 10.1016/j.ejpain.2007.10.003

[56] Zheng Y Zhou Y Wu Q Yue J Ying X Li S Lou X Yang G Tu W Zhou K Jiang S. Effect of electroacupuncture on the expression of P2× 4, GABAA γ 2 and long-term potentiation in spinal cord of rats with neuropathic pain. Brain Res Bull 2020;162:1–10. 32428626 10.1016/j.brainresbull.2020.04.020

